# Artificial Intelligence-based Liver Volume Measurement using Preoperative and Postoperative CT Images

**DOI:** 10.2174/0115734056394257250818060804

**Published:** 2025-08-29

**Authors:** Kwang Gi Kim, Doojin Kim, Chang Hyun Lee, Jong Chan Yeom, Young Jae Kim, Yeon Ho Park, Jaehun Yang

**Affiliations:** 1 Department of Biomedical Engineering, Gachon University College of Medicine, Gil Medical Center, 38-13 Docjeom-ro 3 beon-gil, Namdong-gu, Incheon, 21565, Korea; 2 Department of Health Sciences and Technology, Gachon Advanced Institute for Health Sciences and Technology (GAIHST), Gachon University, Seongnam-si 13120, Korea; 3 Department of Surgery, Gachon University Gil Medical Center, Incheon, Republic of Korea; 4 Department of Bio-health Medical Engineering, Gachon University, Gil Medical Center, Incheon, Republic of Korea

**Keywords:** Liver segmentation, Hepatectomy, Convolutional neural networks, Medical imaging, Computed tomography, Deep learning

## Abstract

**Introduction:**

Accurate liver volumetry is crucial for hepatectomy. In this study, we developed and validated a deep learning system for automated liver volumetry in patients undergoing hepatectomy, both preoperatively and at 7 days and 3 months postoperatively.

**Methods:**

A 3D U-Net model was trained on CT images from three time points using a five-fold cross-validation approach. Model performance was assessed with standard metrics and comparatively evaluated across the time points.

**Results:**

The model achieved a mean Dice Similarity Coefficient (DSC) of 94.31% (preoperative: 94.91%; 7-day post-operative: 93.45%; 3-month post-operative: 94.57%) and a mean recall of 96.04%. The volumetric difference between predicted and actual volumes was 1.01 ± 0.06% preoperatively, compared to 1.04 ± 0.03% at other time points (p < 0.05).

**Discussion:**

This study demonstrates a novel capability to automatically track post-hepatectomy regeneration using AI, offering significant potential to enhance surgical planning and patient monitoring. A key limitation, however, was that the direct correlation with clinical outcomes was not assessed due to constraints of the current dataset. Therefore, future studies using larger, multi-center datasets are essential to validate the model's clinical and prognostic utility.

**Conclusion:**

The developed artificial intelligence model successfully and accurately measured liver volumes across three critical post-hepatectomy time points. These findings support the use of this automated technology as a precise and reliable tool to assist in surgical decision-making and postoperative assessment, providing a strong foundation for enhancing patient care.

## 1 INTRODUCTION

Hepatectomy has been widely performed as a fundamental treatment for various liver diseases [1]. Accurate liver volume measurement is essential to reduce the risk of serious postoperative complications [2]. In particular, the incidence of severe liver dysfunction and infection following hepatectomy is closely related to the volume of the remnant liver [3]. Therefore, accurately measuring the remnant liver volume is crucial for developing hepatectomy plans, predicting the risk of post-hepatectomy liver failure, and evaluating patient prognosis [4]. Although manual volume measurement by clinicians using computed tomography (CT) images is accurate, it is cumbersome, subjective, and time-consuming [5]. In contrast, automated volume measurement methods are objective and rapid; however, their accuracy may decrease depending on the image quality or lesion complexity [6, 7].

To address these issues, research on artificial intelligence (AI)-based automatic liver segmentation for volume measurement has been actively conducted [8-16]. Ahn *et al.* performed liver segmentation for normal livers and various liver diseases (fatty liver, chronic liver disease, cirrhosis, post hepatectomy), achieving a Dice similarity coefficient (DSC) of 97.30, with no significant difference in performance across diseases (p = 0.60) [9]. Farzaneh *et al.* achieved DSCs of 96.13 and 51.21 for liver volume and trauma area segmentation, respectively [10]. Liu *et al.* developed a dense feature selection U-Net model for liver segmentation, achieving a DSC > 94.90 [8]. Christ *et al.* applied conditional random fields (CRFs) post-processing to deep learning model results, achieving a DSC of 94.0 for liver and lesion classification with processing speeds under 100 seconds per volume, saving 24.7 minutes per case, compared with manual volume measurement [14]. Jeong *et al.* conducted liver segmentation and volume estimation for liver transplantation, achieving a DSC of 0.89 and an R2 of 0.99 for volume estimation [16]. Wardhana *et al.* employed a 2.5D model-based convolutional neural network for liver segmentation, achieving a DSC of 0.95 [17]. Takamoto *et al.* performed AI-based volume calculations for patients with liver tumors, achieving a deviation of approximately 1.4% regardless of tumor presence [18]. However, these studies did not address data from different time points where the liver volume varies significantly due to hepatectomy, leaving room for improvement in the generalization performance of liver segmentation.

Previous liver volume measurement studies primarily focused on normal livers, whereas this study assessed a broader range of volumetric changes over time, including measurements taken before liver transplantation or resection, 7 days postoperatively, and 3 months postoperatively. Using the Dice Similarity Coefficient (DSC), we achieved a mean performance score of 0.94, which is comparable to results reported in previous studies. Comparative evaluations were conducted using various performance indicators. This approach enables a comprehensive analysis of liver volume changes before and after surgery and offers a measurement method that can be flexibly applied to livers with significant volumetric variability.

## 2 MATERIALS AND METHODS

This prospective study was conducted from May 31, 2022, through December 31, 2024, to develop and validate an artificial intelligence model for automated liver volume measurement in patients undergoing hepatectomy. The reporting of this diagnostic accuracy study adheres to the STARD 2015 (Standards for Reporting Diagnostic Accuracy Studies) guidelines.

The system used an IBM NVIDIA Tesla V100 GPU (Santa Clara, CA, USA) graphics processing unit. The training model was constructed using a three-dimensional (3D) U-Net architecture to segment the liver region [19]. 3D U-Net is designed for segmenting 3D volume data, enabling high-resolution segmentation and effective processing. The architecture also features skip connections between the encoder and decoder layers. These skip connections enable feature reuse from the encoder at specific resolutions in the decoder, allowing the model to learn high-resolution features more effectively. By using pooling and upsampling layers to reduce and increase the spatial dimensions, the model can learn and reconstruct features at various resolutions.

Fig. (**1**) presents an overview of the training process of the liver segmentation model. CT image data from patients who underwent hepatectomy were preprocessed and divided into training and test datasets. Test data comprised CT images from patients at three time points following hepatectomy (pre-operation, 7 days post-operation, and 3 months post-operation) to verify the model's efficacy in actual procedures. To ensure objectivity in the performance evaluation, five-fold cross-validation was applied during training. Model performance was assessed using the mean and standard deviation of the evaluation metrics obtained per fold. Early stopping was employed to prevent overfitting and to enhance the generalizability of the model. The Adam optimizer was used with a batch size of 4 and a learning rate of 0.001, along with 200 epochs for training [20]. No additional data augmentation was performed. The Dice coefficient loss was used as the loss function for training.

This study utilized CT data from a cohort of 50 patients who underwent hepatectomy at Gil Medical Center. As this was an exploratory study, a formal sample size calculation was not performed. Instead, the sample size was determined prospectively and consecutively, including all eligible patients treated during the study period, resulting in a final cohort of 50 patients. CT images were intended for collection at three time points: preoperatively, 7 days postoperatively, and 3 months postoperatively. The final dataset comprised a total of 114 CT examinations from these 50 patients, distributed as follows: 31 preoperative examinations, 48 examinations at 7 days postoperatively, and 35 examinations at 3 months postoperatively. The number of examinations varies across the time points because not all patients underwent CT scanning at every scheduled follow-up.

Fig. (**2**) visually illustrates the quantitative findings of post-hepatectomy volumetric changes in two representative patients (Samples 1 and 2). For each patient, axial CT images from the three key time points defined in this study are displayed: (a) pre-operative (PreOP), (b) 7 days post-operative (POD7), and (c) 3 months post-operative (POD3mo). The pre-operative images Fig. (**2a**) show the intact liver prior to surgery, while the POD7 images Fig. (**2b**) clearly depict the reduced liver volume immediately following resection. Finally, significant liver regeneration can be observed at the 3-month mark Fig. (**2c**). The red arrows indicate the site of resection and the subsequent area of tissue regrowth. These images provide qualitative evidence of the substantial morphological and volumetric changes the liver undergoes, visually corroborating the volumetric recovery measured by our AI model between POD7 and POD3mo.

Table **1** presents the demographic information of male and female patients participating in this study at each time point (preoperatively, 7 days postoperatively, and 3 months postoperatively). The mean age of male patients at the preoperative time point was 63.0 ± 9.95 years, similar to that of female patients (62.1 ± 8.95 years). The body mass index at the preoperative time point was slightly higher in male patients (25.13 ± 2.95 kg/m^2^) compared to female patients (23.19 ± 3.63 kg/m^2^). Liver function indicators showed postoperative changes compared to preoperative values. Total bilirubin levels tended to decrease slightly 7 days postoperatively but showed an increasing trend at 3 months. Albumin levels were lowest at 7 days postoperatively but showed recovery after 3 months. Among liver enzyme levels, aspartate aminotransferase (AST) and alanine aminotransferase (ALT) levels increased 7 days postoperatively but tended to normalize by 3 months. Notably, ALT exhibited a marked increase 7 days postoperatively in both male (85.51 ± 68.98 U/L) and female (72.89 ± 32.99 U/L) patients. Alkaline phosphatase (ALP) and gamma-glutamyl transferase (GGT) levels showed high interindividual variability, with GGT levels being higher in male patients. At 3 months postoperatively, GGT levels in male patients (132.93 ± 262.75 U/L) were significantly higher than those in female patients (23.71 ± 8.55 U/L). Overall, liver function indicators showed temporary deterioration post-surgery, with most recovering by 3 months. Sex differences were observed, with significant differences in the levels of albumin, AST, and ALT at each time point. Post hoc analysis revealed that albumin levels differed at each time point, while AST and ALT levels showed differences at 7 days and 3 months post-operation.

Selecting an appropriate window width and level value is crucial for distinguishing liver tissues in CT images. The window width represents the range of Hounsfield unit (HU) values displayed in the CT image, whereas the window level indicates the central value of this range. In this study, based on prior empirical knowledge, we set the window width and level to 530 and 250 HU, respectively, for liver segmentation. These values maintain adequate contrast between the liver tissue and surrounding structures, while allowing clear differentiation of the liver parenchyma. Images were resized to 128 × 128 × 128 dimensions.

The deep learning environment used comprisedPython 3.6.2, TensorFlow 1.15.4, and Keras 2.2.5. Model performance was evaluated based on the following metrics: DSC, Specificity, Recall, Precision, and Accuracy. To examine the differences between each time point in demographic information, we conducted Kruskal-Wallis tests. The Shapiro-Wilk test was used to assess whether the data are normally distributed. One-way ANOVA tests were performed to compare the performance differences and volumes at each time point. Additionally, we carried out Tukey's HSD (honestly significant difference) tests as post-hoc analyses. *P* values less than 0.05 were deemed to indicate statistical significance.

## 3 RESULTS

Table **2** presents a detailed comparison of the evaluation metrics for the trained model across the three time points: preoperative (PreOP), postoperative day 7 (POD7), and postoperative 3 months (POD3m). The table includes mean values and 95% confidence intervals for Dice Similarity Coefficient (DSC), Specificity, Recall, Precision, and Accuracy. Statistical analysis (p-value) is also provided to indicate significant differences in metric means across the time points.

All metrics demonstrated high performance across time points (Table **2**); however, statistically significant differences (p < 0.05) were observed for DSC, Recall, Precision, and Accuracy, but not for Specificity (p = 0.53). Specifically, Specificity consistently remained exceptionally high across all time points, with mean values exceeding 99.8%. In contrast, Precision exhibited relatively lower mean values compared to other metrics.

Analyzing the DSC metric, the highest mean DSC value was achieved with preoperative data (94.91% ± 1.72%), followed by POD3m data (94.57% ± 0.77%) and then POD7 data (93.45% ± 1.48%). Similarly, for Precision, the highest mean was observed in PreOP (94.19% ± 2.40%), and the lowest in POD7 (91.50% ± 2.73%). Recall showed a slightly different trend, with POD3m achieving the highest mean value (96.86% ± 1.04%). Accuracy remained consistently high across all time points, with POD7 showing a slightly higher mean (99.86% ± 0.05%).

Fig. (**3**) illustrates liver segmentation performance of the trained model on CT images from a single patient acquired at three time points: preoperative, postoperative day 7 (POD7), and postoperative 3 months (POD3m). Each row corresponds to a specific time point, with columns showing: (a) input CT image, (b) clinically delineated liver segmentation (ground truth), (c) model-generated liver segmentation, (d) false positive (FP) regions of the model prediction (red), and (e) false negative (FN) regions of the model prediction (green).

Table **3** presents the mean ratios of predicted volume to actual volume, expressed as a percentage, and their standard deviations across the three time points: preoperative (PreOP), postoperative day 7 (POD7), and postoperative 3 months (POD3m). The mean ratio for PreOP was 1.01 ± 0.06 (95% CI, 0.98–1.03), increasing to 1.04 ± 0.03 (95% CI, 1.02–1.05) at POD7 and remaining at 1.04 ± 0.02 (95% CI, 1.02–1.04) at POD3m. Statistical analysis revealed significant differences among these time points (p < 0.05).

Fig. (**4**) illustrates three-dimensional liver reconstructions at preoperative, postoperative day 7, and 3-month postoperative time points, visualizing changes in liver morphology and volume during treatment. The preoperative volume was the largest, and the volume on postoperative day 7 was the smallest. The model achieved true positive rates of 90%, 87%, and 92% for preoperative, postoperative day 7, and postoperative 3-month data, respectively, with the lowest true positive rate (87%) observed at postoperative day 7.

Previous studies on liver volume measurement have primarily focused on normal livers. In contrast, this study assessed a broader spectrum of volumetric changes over time, including measurements taken before liver transplantation or resection, as well as at 7 days and 3 months postoperatively. Using the Dice Similarity Coefficient (DSC), we achieved a mean performance score of 0.94, comparable to results reported in prior research. Comparative evaluations were conducted using multiple performance indicators. This approach enables a comprehensive analysis of liver volume changes before and after surgery and provides a measurement method that can be flexibly applied to livers with substantial volumetric variability. At postoperative 3 months (POD3mo), the median percentage volume difference was approximately 3% (3.01%), which visually appears to be the highest median among the three time points, and its IQR is visually the narrowest, suggesting reduced variability at this time point(POD3mo).

## 4 DISCUSSION

This study utilized AI technology to automatically measure liver volume from CT images at three time points (preoperatively, 7 days postoperatively, and 3 months postoperatively) to quantitatively evaluate post-hepatectomy liver regeneration. The results demonstrated statistically significant differences in the predictive performance of the learning model across all metrics (recall, specificity, precision, DSC), except for specificity for the three time points, with generally lower performance for the 7-day postoperative data. Seven days postoperatively, patients were still in the acute recovery phase, which is characterized by various physiological changes, including inflammation, edema, and tissue regeneration in the liver. These rapid changes alter the morphology and density of liver tissue, making it difficult to distinguish clear boundaries, which can negatively impact the predictive performance of the deep learning model [21]. Notably, in terms of volumetric accuracy, the model exhibited the most precise measurements for preoperative data. As shown in Fig. (**5**), the median percentage volume difference for preoperative data was approximately 0% (-0.31%), the closest to zero among all time points, indicating minimal systematic bias in volume prediction before surgery. Furthermore, the mean ratio of predicted to actual volumes for preoperative data was also closest to 1.0 (Table **3**), suggesting the highest volumetric accuracy at baseline.

Liu *et al.* [8] used the U-Net architecture for liver segmentation in abdominal CT images to achieve a DSC of 94.9%. Similarly, Ahn *et al.* [9] achieved a DSC of 0.97 in liver segmentation from abdominal CT images. Christ *et al.*, who applied CRFs postprocessing, achieved a DSC of 94.0 in liver and lesion classification [14]. The results of our study also demonstrated high accuracy, achieving a mean DSC of 0.94 across the three time points, indicating comparable performance to that achieved in other studies, regardless of the time point. Takamoto *et al.* conducted volume calculations using AI for patients with liver tumors, reporting a deviation of approximately 1.4% regardless of tumor presence [18], which is similar to our preoperative volume measurement results [14]. Jeong *et al.* also performed volume estimation, achieving an R^2^ of 0.99 in volume estimation tasks [16]. However, our study is significant as it improves generalization performance in situations with varying liver volumes by training the model on data from different time points, unlike other studies that have focused solely on liver segmentation and volume measurement tasks.

This study demonstrates the capability to automatically measure and assess liver volume changes during post-hepatectomy regeneration, an area not previously addressed in research. Our findings validate the utility of AI technology in liver volume measurement, which is expected to aid in patient monitoring and prognosis prediction after hepatectomy. Personalized treatment strategies could be developed using automatically measured liver volume data to identify patients at high risk of complications early, enabling proactive clinical management. This approach has the potential to enhance the safety and efficacy of liver resection. While the current study establishes the technical accuracy of automated volumetric assessment, a direct investigation correlating AI-predicted volume discrepancies (or volumetric changes) with specific clinical outcomes, such as liver failure, INR elevation, or length of hospital stay, represents a critical next step. A limitation of the current study is that such an analysis was not performed, primarily due to constraints of the existing dataset, including its sample size and the incomplete availability of comprehensive clinical information for all patients. Therefore, future research, ideally leveraging larger, multi-center datasets, will be essential to robustly explore these clinical correlations. Such analyses are vital for translating the demonstrated technical capabilities into effective clinical decision-support tools and for fully realizing the prognostic potential of this AI-driven approach.

## CONCLUSION

In conclusion, this study successfully demonstrates the development and validation of an artificial intelligence model capable of accurately and automatically measuring liver volumes from CT images at three critical time points: preoperatively, at 7 days, and at 3 months post-hepatectomy. Our findings indicate that the model effectively captures the dynamic process of liver regeneration, underscoring its significant potential as a precise tool to support surgical planning, monitor postoperative recovery, and potentially predict patient outcomes.

While the technical accuracy of the AI-driven volumetric assessment is promising, we acknowledge certain limitations. The primary focus of this study was the development and validation of the segmentation model’s performance across different time points. A direct and comprehensive investigation into the correlation between AI-predicted volumetric changes and specific clinical outcomes, such as post-hepatectomy liver failure, INR elevation, or length of hospital stay, was not conducted. This was mainly due to constraints of the current dataset, including its sample size and the incomplete availability of uniformly collected, comprehensive clinical outcome data necessary for such robust correlative analysis. Therefore, future research should prioritize several key areas. Firstly, validation of the model's performance and generalizability using larger, more diverse, and ideally multi-center datasets is essential. Secondly, and critically, future studies must focus on rigorously investigating the correlation between the AI-derived volumetric measurements and a range of clinical outcomes. Such analyses, leveraging appropriately rich datasets, are imperative to translate the technical capabilities demonstrated in this work into clinically impactful decision-support tools. Successfully addressing these aspects will be crucial for fully realizing the prognostic potential of automated liver volumetry and integrating this technology into routine clinical practice to improve patient care following hepatectomy.

## Figures and Tables

**Fig. (1):**
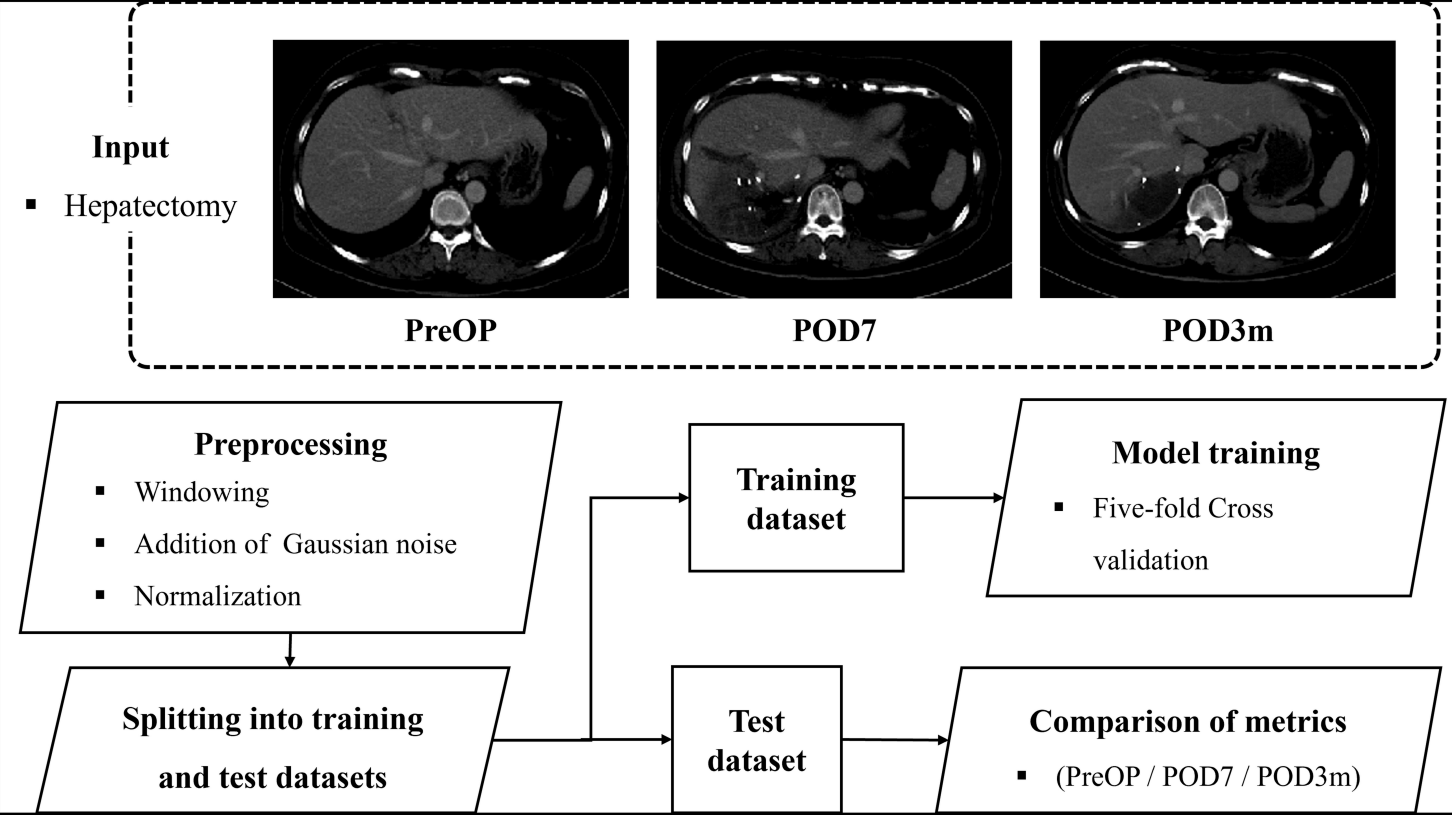
Schematic overview of the liver segmentation model training process: preprocessed data is divided into training and test sets for model training and validation on patients undergoing hepatectomy.

**Fig. (2):**
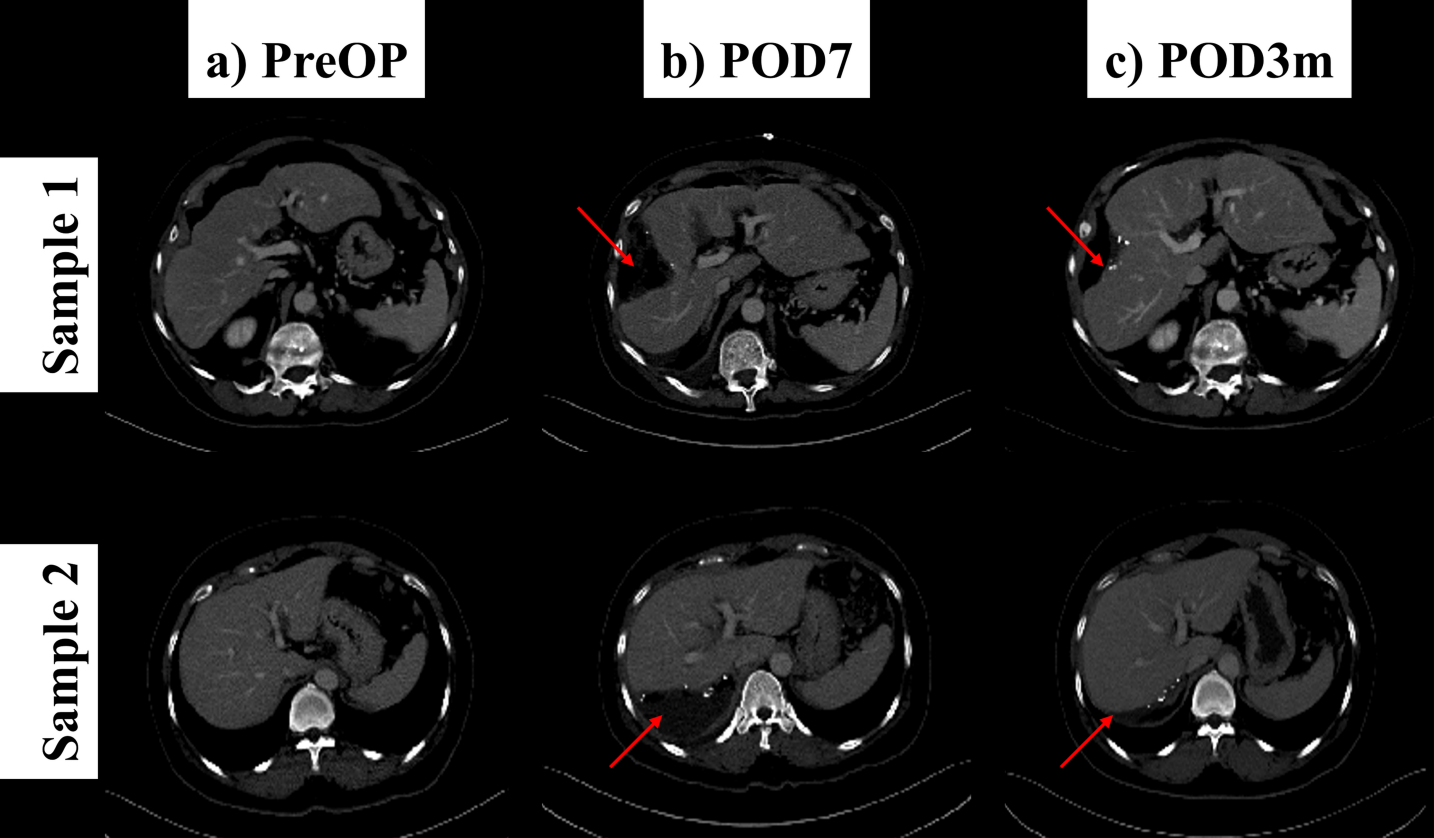
This figure depicts representative axial CT images demonstrating liver volume changes (Red arrows) following hepatectomy from two different patients (Samples 1 and 2) at three distinct time points: (**a**) Pre-operative (PreOP), (**b**) 7 days post-operative (POD7), and (**c**) 3 months post-operative (POD3mo).

**Fig. (3):**
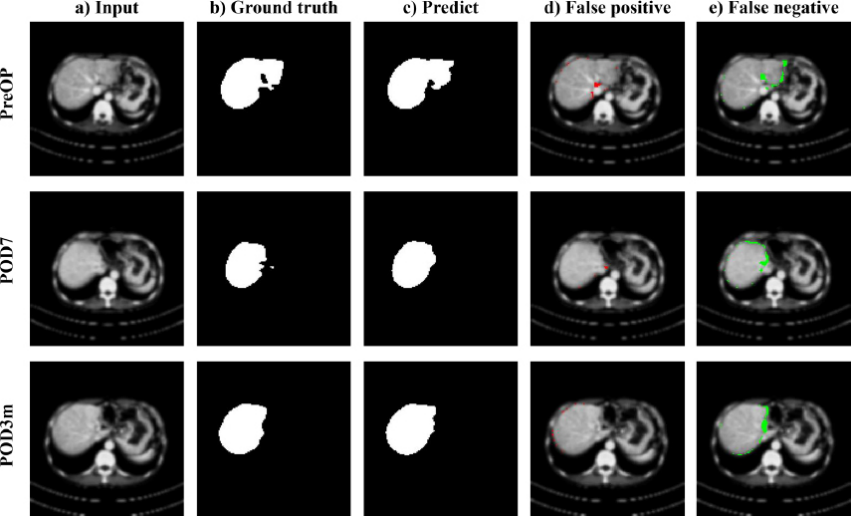
(**a**) Input CT image, (**b**) Clinically segmented ground truth region, (**c**) Model-predicted region, (**d**) False positive regions of model prediction (red), (**e**) False negative regions of model prediction (green). Each row corresponds to the time points of preoperative, postoperative day 7, and postoperative 3 months (POD3m).

**Fig. (4):**
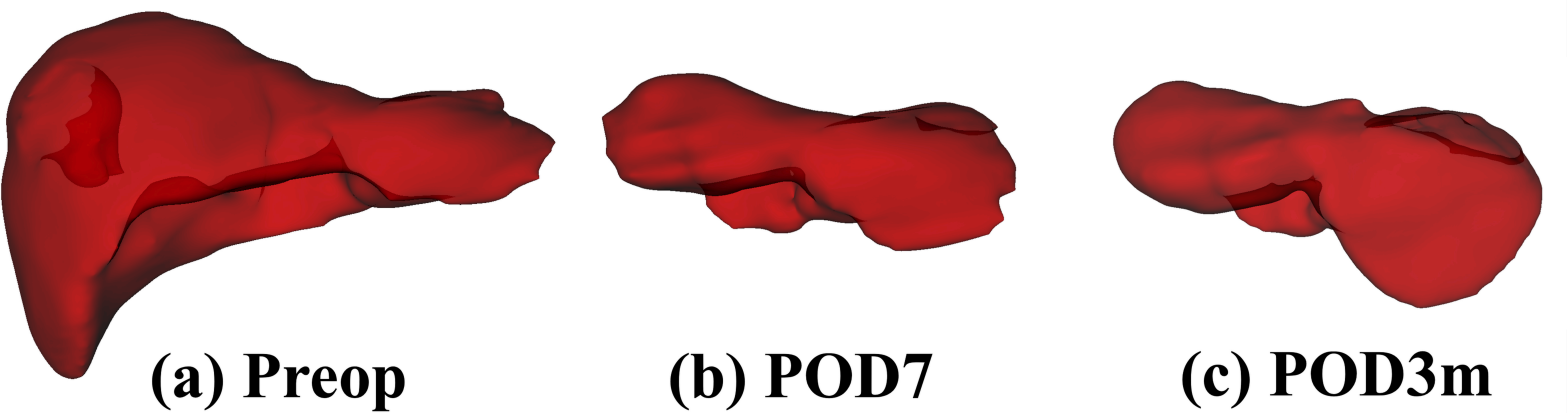
Three-dimensional visualization of liver volume at different time points. (**a**) Preoperative state; (**b**) 7 days postoperative; (**c**) 3 months postoperative.

**Fig. (5):**
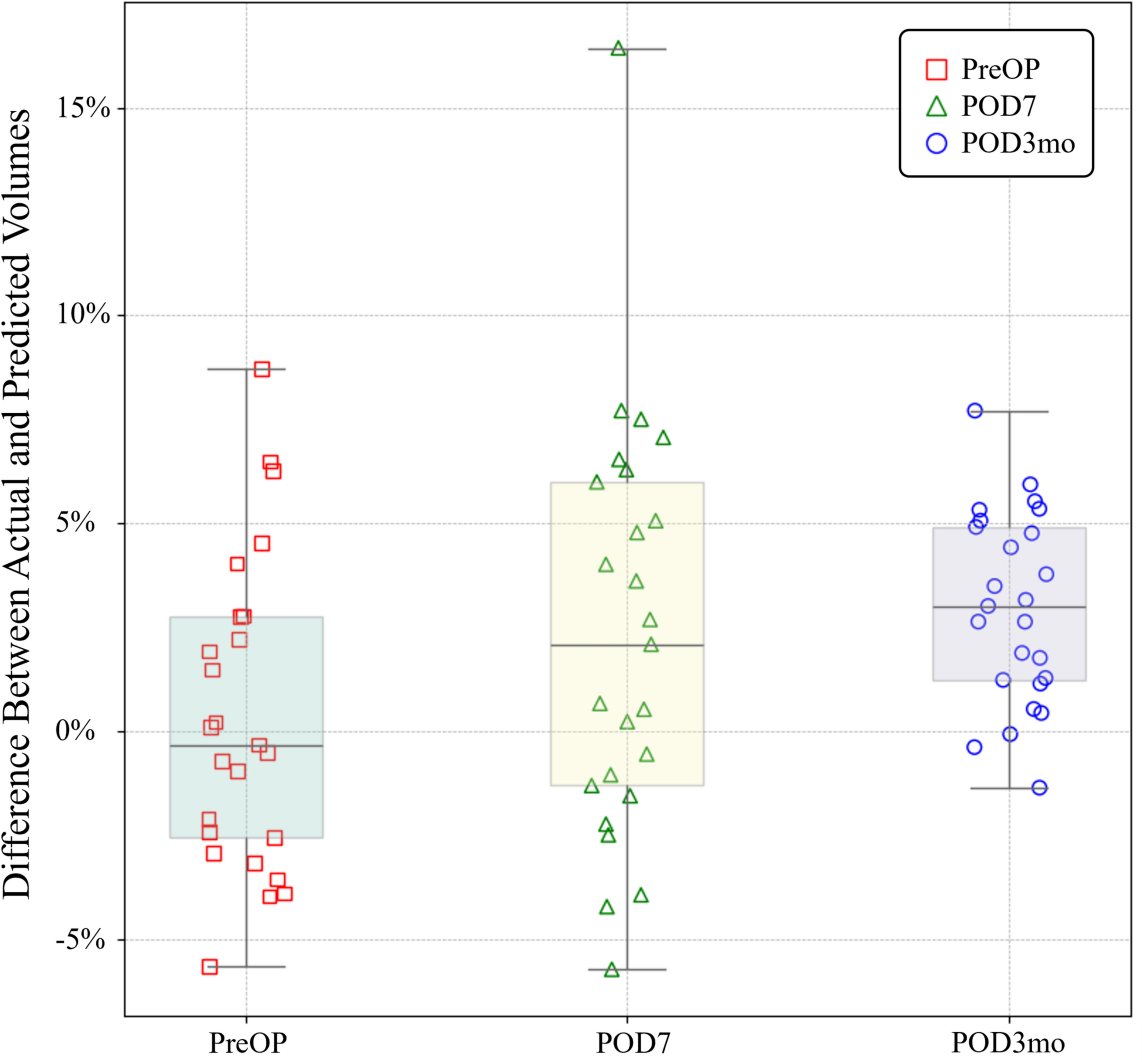
Box plot of the difference in liver volume between the actual and predicted masks for a single patient across three time points following hepatectomy.

**Table 1:** Demographic and clinical characteristics of study participants at three perioperative time points, stratified by sex. All values are presented as mean ± standard deviation, with the 95% confidence interval shown in parentheses.

**Category**	**Mean**±STD **(95% Confidence interval)**						***p*-val**
	**PreOP**		**POD7**		**POD3m**		
	**Male** **(n=40)**	**Female** **(n=10)**	**Male** **(n=38)**	**Female** **(n=9)**	**Male** **(n=38)**	**Female** **(n=9)**	
Age	63.0±9.95 (59.8-66.1)	62.1±8.95 (57.5-70.4)	63.2±10.1 (59.9-66.6)	64.0±8.5 (58.6-71.8)	62.0±10.3 (59.6-66.1)	63.6±9.2 (57.9-71.8)	0.94
Body Mass Index (kg/m^2^)	25.13±2.95 (24.3-26.2)	23.19±3.63 (20.7-25.7)	25.49±3.45 (24.1-26.5)	24.25±2.23 (22.4-26.0)	25.26±2.23 (18.7-31.5)	23.14±0.00 (23.1-23.1)	0.88
Total bilirubin	0.92±0.50 (0.75-1.05)	0.77±0.30 (0.49-0.92)	0.75±0.28 (0.65-0.84)	0.65±0.19 (0.49-0.80)	0.98±0.48 (0.81-1.12)	0.62±0.28 (0.39-0.81)	0.18
Albumin	4.34±0.37 (4.22-4.44)	4.23±0.39 (3.83-4.50)	3.34±0.40 (3.21-3.47)	3.11±0.26 (2.90-3.31)	4.17±0.39 (3.14-7.29)	4.16±0.13 (3.78-4.30)	<0.05
Aspartate Aminotransferase	41.32±36.08 (30.3-52.62)	39.43±20.66 (23.4-50.3)	45.86±23.93 (40.1-58.0)	44.67±24.26 (24.8-64.4)	34.61±30.33 (22.8-40.6)	25.71±4.30 (21.8-28.5)	<0.05
Alanine Aminotransferase	40.23±31.68 (29.7-48.7)	35.00±29.02 (14.5-51.8)	85.51±68.98 (67.0-113.3)	72.89±32.99 (45.9-99.7)	26.57±18.04 (19.2-30.1)	20.71±7.02 (14.1-24.9)	<0.05
Alkaline Phosphatase	90.45±30.80 (78.6-98.2)	100.29±62.16 (59.9-140.0)	95.34±34.08 (86.7-110.1)	149.78±119.40 (52.4-247.1)	121.39±101.92 (85.0-147.6)	93.29±33.83 (70.0-123.2)	0.26
Gamma-Glutamyl Transferase	87.81±76.07 (62.9-110.2)	39.71±36.44 (14.9-64.2)	73.43±53.52 (61.1-99.1)	105.78±126.17 (2.9-208.6)	132.93±262.75 (32.6-185.8)	23.71±8.55 (18.7-32.3)	0.27

**Table 2:** Evaluation metrics (recall, specificity, precision, accuracy, DSC: dice similarity coefficient) of the trained model on CT images pre-operation, 7 days post-operation, and 3 months post-operation.

-	**PreOP**	**POD7**	**POD3m**	***p*-val**
Dice Similarity Coefficient (95% confidence interval)	94.91±1.72 (94.62-95.21)	93.45±1.48 (93.01-93.90)	94.57±0.77 (94.40-94.74)	<0.05
Specificity (95% confidence interval)	99.89±0.00 (99.87-99.91)	99.90±0.00 (99.88-99.92)	99.86±0.00 (99.88-99.90)	0.53
Recall (95% confidence interval)	95.70±0.02 (95.12-96.28)	95.57±2.14 (94.80-96.35)	96.86±1.04 (96.62-97.10)	<0.05
Precision (95% confidence interval)	94.19±2.40 (93.16-95.22)	91.50±2.73 (90.23-92.77)	92.39±1.16 (91.98-92.28)	<0.05
Accuracy (95% confidence interval)	99.82±0.03 (99.91-99.83)	99.86±0.05 (99.85-99.97)	99.85±0.04 (99.84-99.86)	<0.05

**Table 3:** Mean ratios of predicted to actual volumes (with standard deviations and 95% confidence intervals) and p-value from mean comparison test across time points. Results for the three categories (pre-operation, 7 days post-operation, and 3 months post-operation).

-	**PreOP**	**POD7**	**POD3m**
Percentage (%) (95% confidence interval)	1.01±0.06 (0.98-1.03)	1.04±0.03 (1.02-1.05)	1.04±0.02 (1.02-1.04)
*p*-val	<0.05		

## Data Availability

The datasets generated and analyzed during the current study are not publicly available due to privacy concerns and institutional policy; however, they are available from the corresponding authors [K.K] and [D.K] upon reasonable request, provided that appropriate institutional approval is obtained.

## LIST OF ABBREVIATIONS

PreOP: Preoperative
POD7: Postoperative day 7
POD3m: Postoperative 3 months
CT: Computed tomography
IQR: Interquartile range

## STANDARDS OF REPORTING

STARD guidelines were followed.
